# Effect of Mesoporous Nano Water Reservoir on MR Relaxivity

**DOI:** 10.1038/s41598-017-11710-2

**Published:** 2017-09-11

**Authors:** Palani Sharmiladevi, Viswanathan Haribabu, Koyeli Girigoswami, Abubacker Sulaiman Farook, Agnishwar Girigoswami

**Affiliations:** 1 Faculty of Allied Health Sciences, Chettinad Hospital and Research Institute (CHRI), Chettinad Academy of Research & Education (CARE), Kelambakkam, Chennai, 603 103 India; 2 Department of Radiology, Chettinad Hospital and Research Institute (CHRI), Kelambakkam, Chennai, 603 103 India

## Abstract

In the present work, an attempt was made to engineer a mesoporous silica coated magnetic nanoparticles (MNF@mSiO_2_) for twin mode contrast in magnetic resonance imaging (MRI) with reduced toxicity. Superparamagnetic manganese ferrite nanoparticles were synthesized with variable mesoporous silica shell thickness to control the water molecules interacting with metal oxide core. 178 nm was the optimum hydrodynamic diameter of mesoporous ferrite core-shell nanoparticles that showed maximum longitudinal relaxation time (T1) and transverse relaxation time (T2) in MRI due to the storage of water molecules in mesoporous silica coating. Besides the major role of mesoporous silica in controlling relaxivity, mesoporous silica shell also reduces the toxicity and enhances the bioavailability of superparamagnetic manganese ferrite nanoparticles. The *in vitro* toxicity assessment using HepG2 liver carcinoma cells shows that the mesoporous silica coating over ferrite nanoparticles could exert less toxicity compared to the uncoated particle.

## Introduction

Biomedical imaging is presently the most widely used tool for diagnosis and detection of several diseased conditions. Among these imaging modalities, molecular imaging is found to be important for the diagnosis of cancer^1, 2^. Magnetic resonance (MR) imaging has gained interests from many researchers due to its fascinating ability in diagnosis and staging of disease conditions^3^. There are many agents developed in order to enhance the image contrast between normal and affected tissue based on the T1 and T2 relaxation properties. T1 relaxation corresponds to the time that takes for the longitudinal component of magnetization to recover 1–1/e of its initial value where as T2 corresponds to the time that takes for the transverse component of magnetization to recover 1/e of its initial value (‘e’ is the base of natural logarithm). A very well-known class of T1 contrast agents are gadolinium chelate complexes which interact with the water molecules thereby increasing the T1 relaxivity^4^. This increase in T1 relaxivity leads to enhanced MR signal in a T1 weighted image. Similarly, a T2 contrast agent namely iron oxide nanoparticles increases T2 relaxivity leading to negative contrast in T2 weighted MR image^5^. Hence it is important to understand that relaxivity and relaxation properties of a contrast agent plays a major role in enhancing contrast of an MR image leading to better diagnosis.

Magnetic nanoparticles are used by several researchers as contrast agents in MR imaging^6, 7^. More recently, research interests have grown in developing contrast agents with dual mode MR imaging i.e. T1 and T2 contrast imaging with single imaging agent. This concept of dual mode MRI is found to have better and early diagnostic feature of certain types of diseases which may be missed when done in single mode^8^. T1 shortening agents such as gadolinium and manganese can be doped with T2 shortening agent such as iron oxide nanoparticles using simple chemical routes, like co-precipitation method. This core-shell structure of magnetic nanoparticle shows superparamagnetic nature thereby enhancing image contrast in both T1 and T2 images^9^. Our previous study focused on the optimization of manganese doped iron oxide nanoparticles entrapped in dendrimers for dual contrasting role in MR imaging^10^.

It is hypothesized that the MR relaxivity for these recently developed dual mode MR contrast agents depends on the interaction of water molecules. Many studies show that coating the magnetic nanoparticles with mesoporous silica helps in enhancing the image contrast^11^. Moreover, mesoporous silica coating on magnetic nanoparticles makes these nanoparticles biocompatible and highly stable ensuring longer circulation time when injected into the biological system^12^. With concurrence to our previous study, the present study concentrates on the interaction of reservoir water molecules entrapped in the mesoporous silica coating of the magnetic nanoparticles to identify the effect of mesoporous silica shell thickness on MR relaxivity. This variation in MR relaxivity due to mesoporous silica shell in turn will enhance MR image contrast in both T1 and T2 MR images.

## Results and Discussion

The present study was carried out using co-precipitation method where manganese ferrite nanoparticles (MNF) was synthesized and mesoporous silica coating with 6 variable shell thicknesses were done on the MNFs applying six varying concentrations of TEOS (0.047, 0.094, 0.188, 0.282, 0.376 and 0.470 gm). The resulting MNF@mSiO_2_ with variable shell thickness were marked as S1, S2, S3, S4, S5, and S6 respectively. Characterization at each step of the synthesis process was performed using the following methods.

**Figure 1:**
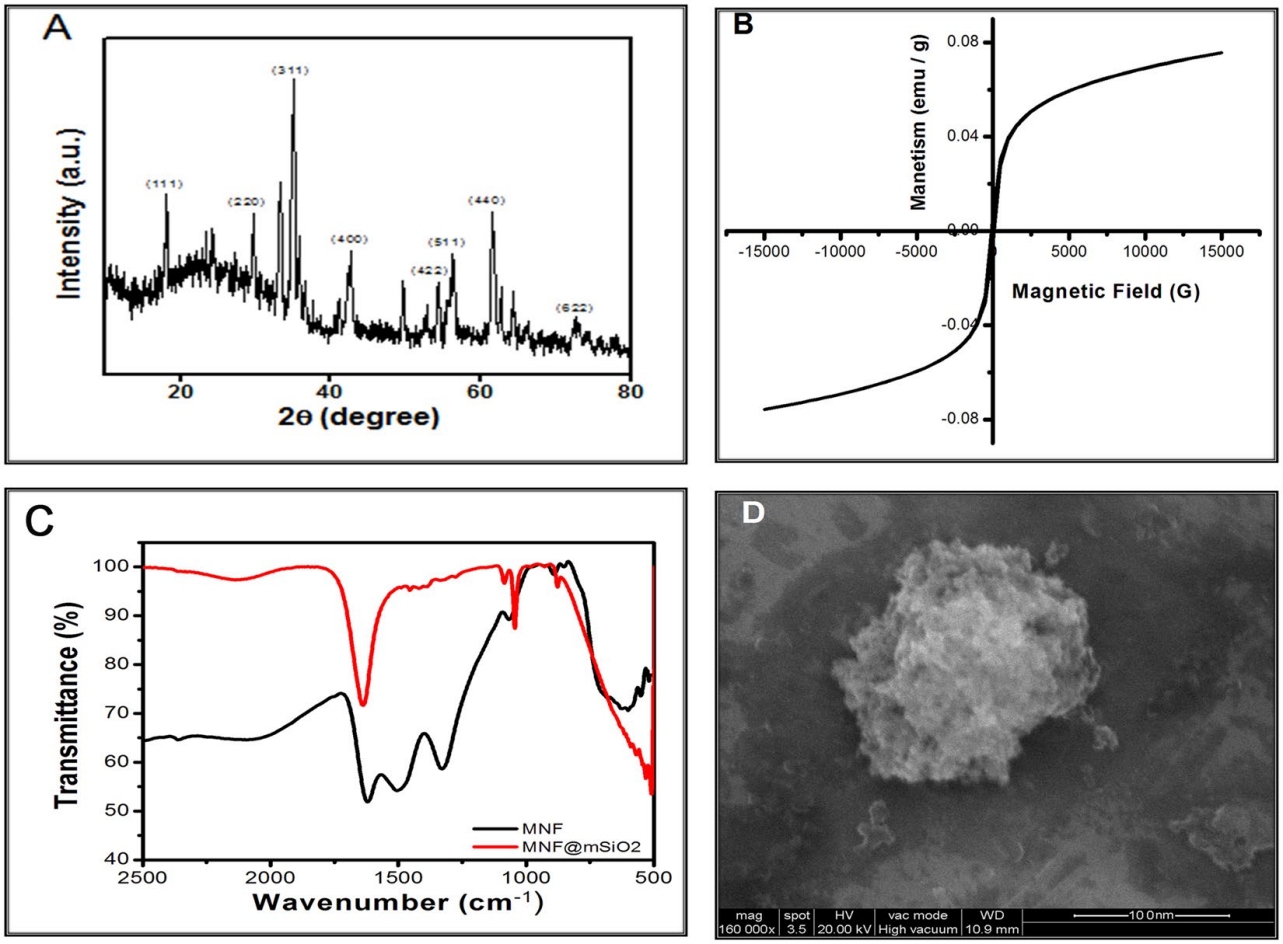
(**A**) X-ray diffraction pattern of synthesized MNF using Co-precipitation method. (**B**) Hysteresis loop of MNF at room temperature for the assessment of superparamagnetism. (**C**) FTIR spectra of MNF & MNF@mSiO_2_. (**D**) FESEM image of mesoporous silica coated nanoparticles (S3).

Crystals of the synthesized MNFs were analysed using X-ray diffraction method to measure the crystal size, lattice parameters and structural morphology. The average diameter and crystallinity were analysed using characteristic peaks at specific 2θ scales (Fig. 1A). The mean diameter of the prepared MNF was calculated using Debye Scherrer’s formula:

$$D=\frac{k\lambda }{\beta \,\cos \,\theta }$$

Where D is the crystalline size of the particle, β is the full width at half maxima (FWHM) in radians at the strongest reflection of XRD pattern, and θ is the Bragg’s angle. K is the Scherrer constant with the value of 0.89 and λ = 0.154178 nm is the wavelength of radiation. The crystal size was determined to be 12.73 nm.

Inductively Coupled Plasma Optical Emission Spectrometry (ICP-OES) was applied to measure the percent concentration of iron & manganese in all the engineered nanoparticles. Mn was determined at 257.610 nm and 279.482 nm where as Fe was determined at 238.2 nm and 259.94 nm. Mn:Fe was calculated using the data obtained and it was 1:2 for all the MNF and mesoporous silica coated MNF nanoshell particles (MNF@mSiO_2_).

Vibrating sample magnetometer (VSM) was utilized to evaluate the superparamagnetic nature of the synthesized MNF at room temperature. The absence of hysteresis loop in Fig. 1B indicates that the synthesized MNF has superparamagnetic behaviour. The saturation magnetization (M_S_) for the MNF was determined to be 75 emu/g, indicating the presence of single domain structure in MNF. The results also confirm the superparamagnetic nature of the MNF that will support in MR imaging.

FTIR spectra of the synthesized MNF and MNF@mSiO_2_ are shown in Fig. 1C which is considered as an appropriate technique to confirm the formation of nanoshells with MNF coated with mesoporous silica as shell material. The spectra were acquired in transmittance mode where the presence of characteristic band at 529 cm^−1^ for MNF corresponds to Fe-O bonds. This band is found to be shifted to 533 cm^−1^ after coating with mesoporous silica shell. Furthermore, C=O stretching band noted at 1634 cm^−1^ in MNF is found to have shifted to 1645 cm^−1^ after mesoporous silica coating confirming the formation of MNF and MNF@mSiO_2_ nanoshell. The band noted at 1085 cm^−1^ is characteristic of Si-O-Si stretching which is noted in MNF@mSiO_2_ that highly supports the formation of MNF@mSiO_2_ nanoshell. These characteristic bands confirm the synthesis of MNF@mSiO_2_.

The surface morphology and size of S3 were determined by scanning electron microscopy too (Fig. 1D). It shows spherical structure with rough surface which indicates the mesoporous silica coating. The size of the S3 MNF@mSiO_2_ is approximate 141 nm.

### Determination of Colloidal nature

**Figure 2:**
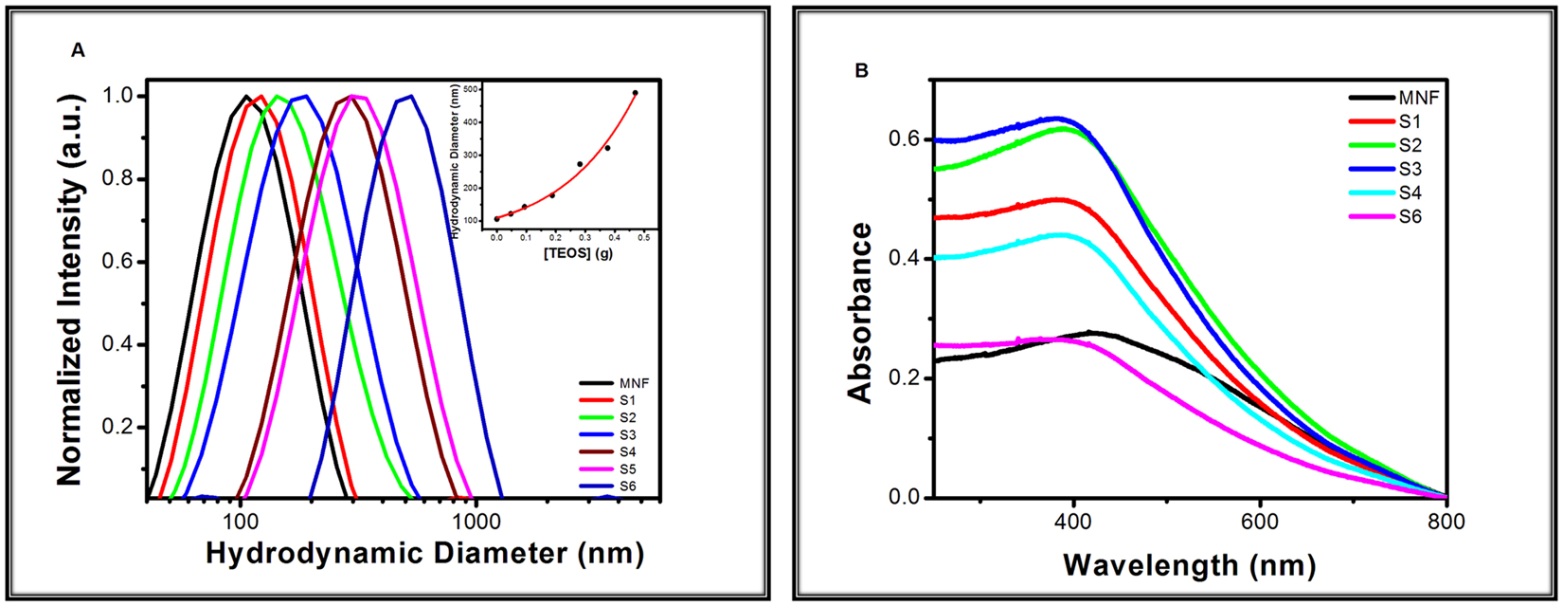
(**A**) Spectral representation of hydrodynamic diameter of colloidal MNF and different MNF@mSiO_2_. Inset shows variations in hydrodynamic diameter with increasing concentrations of TEOS. (**B**) Absorption spectra of colloidal MNF and MNF@mSiO_2_.

Colloidal nature of the synthesized MNF and MNF@mSiO_2_ was determined by identifying the hydrodynamic diameter of the nanomaterials using dynamic light scattering (DLS) technique. Figure 2A shows the changes in hydrodynamic diameter (dH) and the colloidal nature of the synthesized nanomaterials. It is noted that the hydrodynamic diameter increases when MNF is coated with mesoporous silica and furthermore, as the concentration of the TEOS increases the hydrodynamic diameter also increases. The dHs were recorded for MNF, S1, S2, S3, S4, S5 and S6 as 106, 122, 143, 178, 273, 322, and 490 nm respectively. The relation between TEOS concentration and hydrodynamic diameter is plotted in the inset of Fig. 2A to show the nonlinearity in the dH increment. This increase in hydrodynamic diameter confirms the different shell thickness outside the colloidal MNF.

### Spectrophotometric observation

UV-visible spectrophotometer was utilized to determine the absorption spectra of the prepared MNF and MNF@mSiO_2_. Figure 2B shows the absorption spectra that were recorded for MNF and MNF@mSiO_2_ prepared with increasing concentration of TEOS. The wavelength at which the absorption maxima or plasmon bands were noted and their corresponding intensities are tabulated in Table 1. The citrate stabilized MNF showed a broad plasmon band at 428 nm. On coating with TEOS of varying concentrations as shown in Table 1, a blue shift from 428 nm to 380 nm was observed in the plasmon band with increased absorbance. This blue shift clearly indicated that the thickness of mesoporous silica coating has direct impact on the absorption of MNF. On further increase of silica shell thickness, the plasmon band shifted back towards the initial position with a reduction in absorbance.

**Table 1:** Plasmon band for MNF@mSiO_2_ for variable TEOS concentration.

@Si gm	Plasmon band (nm)	Intensity (a.u.)
MNF	428	0.28
S1	389	0.50
S2	386	0.62
S3	380	0.66
S4	390	0.44
S6	406	0.26

The plasmon band noted at 428 nm for citrate stabilized MNF (C-MNF) can be attributed to the d-d transitions of Mn in MNF nanoparticles, as the degeneracy of the Eg ground state term of d4 in a high-spin octahedral environment has been lifted by the Jahn–Teller effect as per ligand field theory (LFT). It was also noted that the optical density (OD) of the MNF@mSiO_2_ increased with initial blue shift with increase in silica shell thickness, reached a maxima and finally exhibited red shift with decrease in OD towards the C-MNF.

The shift in the spectra may be attributed to the presence of oxygen containing silica or water molecules. The red shift in the spectra after the hydrodynamic diameter of 178 nm of the prepared nanoshells clears that silica is not the sole reason for the spectral shift. Hence, the possible reason for the spectral shift might be due to the presence of water molecules residing in the mesoporous silica that interacts with metal-oxide core. The water molecules had clear access to the metal-oxide core till 178 nm dH after which the access was limited for the water molecules to interact with the metal-oxide core. Therefore, water molecules stored in the mesoporous silica as reservoir plays a major role in the spectroscopic character of MNF@mSiO_2_.

### Phantom MR imaging studies

**Figure 3:**
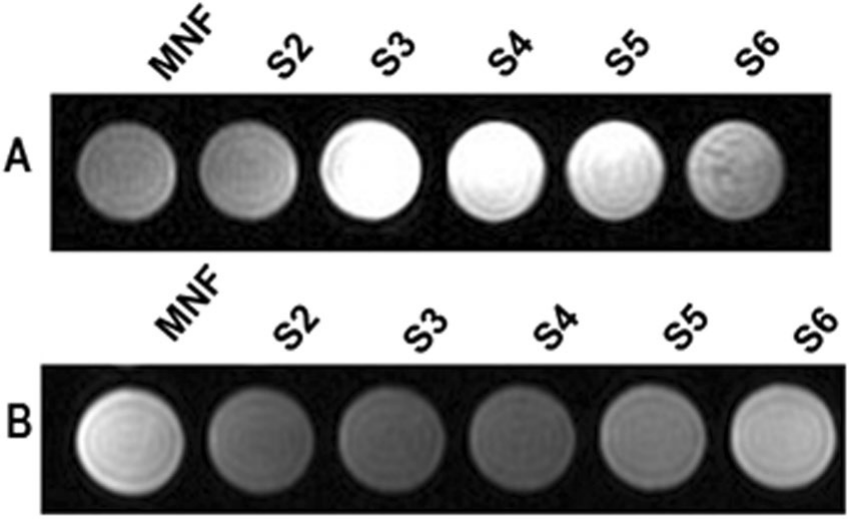
MR phantom images for different MNF@mSiO_2_. (**A**) T1 weighted and (**B**) T2 weighted MRI.

Phantom MR imaging studies were performed to identify the optimal mesoporous silica shell thickness that could help in achieving better MR image contrast. Figure 3 shows the T1 and T2 weighted MR images respectively acquired with variable shell thicknesses in MNF@mSiO_2_. In case of T1 weighted imaging, it was noted that the intensities increased with increasing silica shell thicknesses till S3 and decreased with further increase in shell thicknesses. On the other hand, T2 weighted imaging showed decrease in intensities till S3 beyond which the intensities were found to increase. S4 and S3 show almost same intensity with little variation. All the intensities are tabulated in Table 2. These results from Phantom MR imaging supported the results noted with UV-Visible absorption spectra where the interaction of water molecules residing in mesoporous silica shell with manganese was higher for S3. Furthermore, it was noted that the enhancement in image contrast was not optimal for other silica shell thicknesses above S3 due to the distance between the water molecules and the particle core. Hence it was concluded that the enhancement of image contrast depends on the water reservoir in the mesoporous silica shell. Based on these results, all further experiments were conducted with MNF@mSiO_2_ of hydrodynamic diameter of 178 nm which was prepared with 0.188 g of TEOS.

**Table 2:** Tabulated intensities obtained from T1 and T2 weighted phantom MR images.

Particles	T1 Intensity (a.u.)	T2 Intensity (a.u.)
MNF	81	178
S2	89	91
**S3**	**192**	**78**
S4	168	81
S5	137	122
S6	97	177

### Determination of MR relaxivities

**Figure 4:**
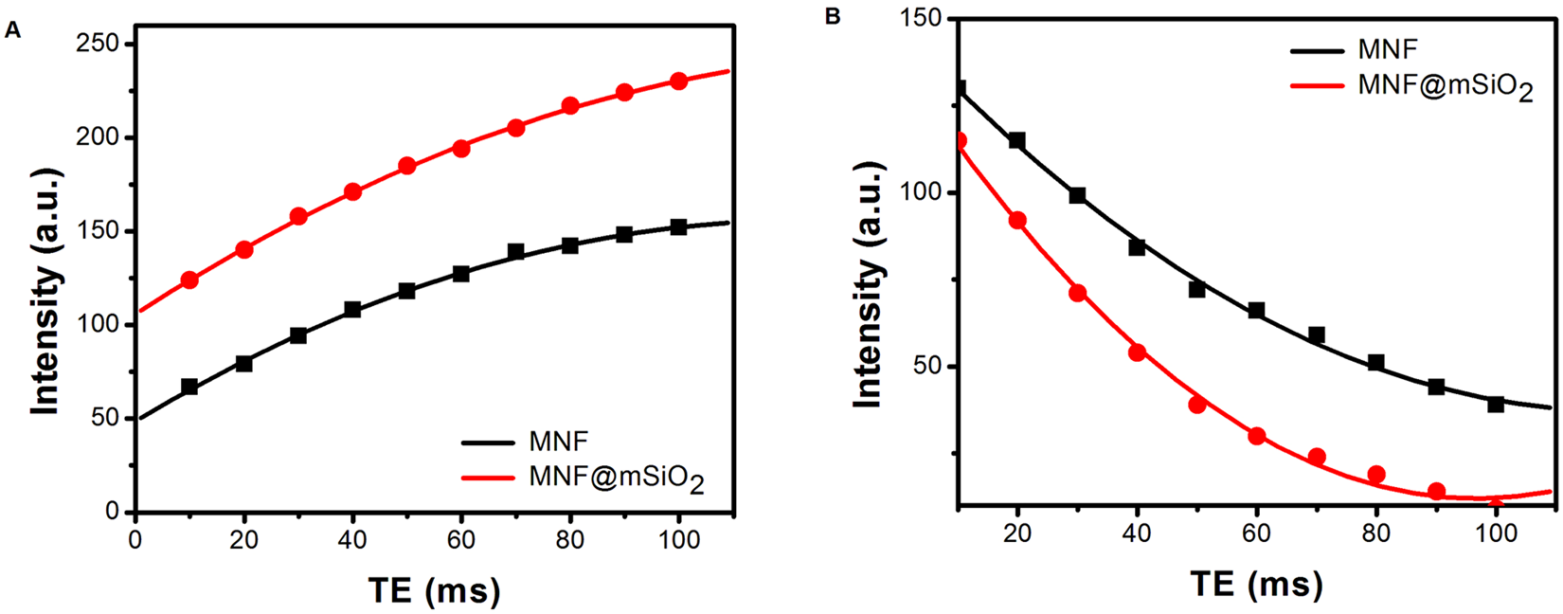
(**A**) Exponential growth curve for T1 signal intensity with increasing Echo Time. (**B**) Exponential decay curve for T2 signal intensity with increasing Echo Time.

TE is the time at which the electrical signal induced by the spinning protons is measured. Therefore, T1 and T2 relaxation properties were determined for MNF and MNF@mSiO_2_ by using variable TE for acquiring T1 and T2 weighted images and intensities were detected. Figure 4A,B show the exponential growth curve for T1 signal intensities and exponential decay curve for T2 signal intensities with variable TE respectively for MNF and MNF@mSiO_2_. The T1 relaxation for MNF and MNF@mSiO_2_ was determined as 425 ms and 138 ms respectively. On the other hand, the T2 relaxation was determined to be 75 ms and 35 ms for MNF and MNF@mSiO_2_ respectively.

These T1 and T2 relaxation values were used to calculate the longitudinal relaxivity (r1 = 1/T1) and transverse relaxivity (r2 = 1/T2), the parameters describing the efficacy of an image contrast agent. The r1 and r2 values for MNF and MNF@mSiO_2_ were found to be 0.00235, 0.013 and 0.00724, 0.03 respectively. In order to identify the potential application of these nanoshells as dual contrast MR imaging agent, it is necessary to calculate the r1/r2 ratio. Previous studies have shown that higher r1/r2 ratio ( > 10) supports T2 contrast and lower r1/r2 ratio (1–3) supports T1 contrast. If the values are intermediate, then it can be concluded that the agent can support in dual contrast MR imaging^5^. The r1/r2 ratio for MNF and MNF@mSiO_2_ was identified to be 5.5 and 4.14 respectively. This shows that both MNF and MNF@mSiO_2_ possess dual contrasting ability in MR imaging. Since the value 4.14 for MNF@mSiO_2_ is near to the limiting value of T1 contrast, it can be concluded that MNF@mSiO_2_ has twin mode contrast ability in MRI with better T1 effect.

### Cell viability assessment

**Figure 5:**
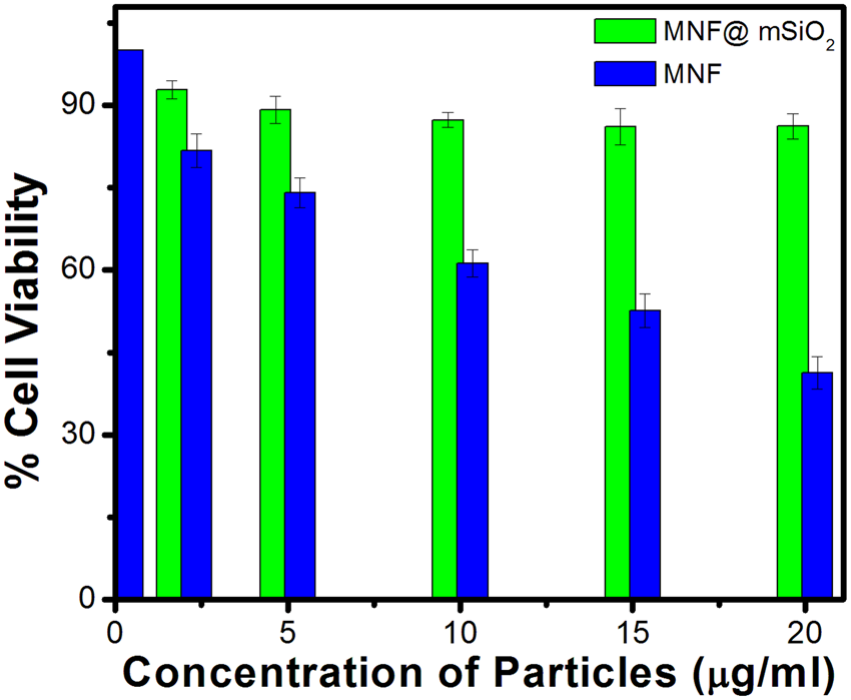
Graphical representation of percent cell viability obtained from MTT assay using HepG2 liver carcinoma cells.

Assessment of toxicity for the MNF and MNF@mSiO_2_ used in the present experiment was conducted using HepG2 liver carcinoma cells by MTT assay. Figure 5 is a graphical representation of percent cell viability of HepG2 liver carcinoma cells for increasing concentrations of MNF and MNF@mSiO_2_ using MTT assay. The results show that the percentage of cell viability for MNF@mSiO_2_ is higher than that noted for MNF. Hence it can be concluded that MNF@mSiO_2_ nanoshells are less toxic than MNF used without any coating.

It is concluded that mesoporous silica coated Mn doped iron oxide nanoshells can be used as dual contrast MR imaging agent. The water molecules that get stored in mesoporous silica shell as reservoir can help in enhancing the T1 contrast due to interaction with Mn in the MNF. An optimal reservoir for water molecules to be stored can be achieved with 178 nm hydrodynamic diameter of MNF@mSiO_2_. This was confirmed using phantom MR imaging and calculation of r1/r2 ratios. Furthermore, MNF@mSiO_2_ enhances T1 contrast in MR imaging due to reduced distance between the interacting water molecules and Mn. Toxicity assay conducted with HepG2 liver carcinoma cells shows that MNF@mSiO_2_ has better percentage of cell viability compared to MNF used without any coating. All these results show that mesoporous silica coated manganese doped iron oxide nanoshells can be used as a potential dual contrasting MR imaging agent for early diagnosis.

## Methods

### Synthesis of manganese ferrite nanoparticles

Synthesis of MNF was carried out based on previously reported procedure with minor modifications^10^. Briefly, chloride salts of ferric and manganese at the molar ratio of 0.5 was used in the synthesis of MNF. Citric acid was added to these ionic solutions as a stabilizing agent. Oxidising agent used in the procedure was 6 M sodium hydroxide solution which was added with the ionic solutions at the rate of 1 drop per second with constant mechanical stirring. The mixture was allowed to stir for an hour after complete addition of the ionic solution. The final solution was then centrifuged at 3000 rpm for 10 min and the pellet was redispersed in distilled water. The centrifugation process was repeated 3–4 times and the resulting pellet was dispersed in water for further modifications and studies^10^.

### Synthesis of mesoporous silica coated MNF nanoshell particles

Mesoporous silica coated MNF nanoshell particles were synthesized following the protocol of Zhang *et al*., with slight modifications^13^. About 2.5 mL of the prepared MNF was dispersed in 10 mL of 0.1 M hydrochloric acid by sonication for 10 min. The solution was centrifuged at 3000 rpm for 10 min and the particles were dispersed in a solution containing ethanol and distilled water in the ratio of 4:1 and 0.17 g CTAB dissolved in it. The mixture was vigorously stirred for 30 min and then 0.75 mL of aqueous ammonia solution and 50 µL TEOS were added.

The resulting mixture was stirred continuously for 12 h to enable efficient coating of mesoporous silica over the magnetic nanoparticles. After stirring, the final solution was centrifuged and washed with ethanol for 3–4 times and finally with water. CTAB in mesoporous silica was removed by dispersing the particles in ethanolic solution of ammonium nitrate and stirring for 4 h^8^. The resulting solution was stirred at 60°C for 15 min, centrifuged and washed with ethanol before final dispersion in water.

The above procedure was repeated with six (0.047, 0.094, 0.188, 0.282, 0.376 and 0.470 gm) varying concentrations of TEOS to get resulting MNF@mSiO_2_ with variable shell thickness and were marked as S1, S2, S3, S4, S5, and S6 respectively.

### Phantom MR imaging

The synthesized MNF@mSiO_2_ nanoshells with variable shell thickness of mesoporous silica were taken in a 24 well plate in the increasing order. The plate was then imaged under MRI scanner with both T1 and T2 weighted imaging protocols mentioned in our previous work^10^. Using Centricity software intensities of the resulting images were determined. These identified intensities were then plotted against varying mesoporous silica shell thicknesses. The r1 and r2 were calculated by acquiring T1 FLAIR and T2 Turbo Spin Echo sequences with variable echo times (TE) and r_1_/r_2_ ratio was determined to conclude on the dual contrasting ability of the synthesized MNF and MNF@mSiO_2_ nanoshells^10^.

### Cytotoxicity assessment using HepG2 liver carcinoma cells

Cytotoxicity assessment for the synthesized MNF and MNF@mSiO_2_ was conducted by using HepG2 hepatocellular carcinoma cells. MTT Assay to identify the cell viability was performed following a standard protocol^14^.

Bruker Alpha FTIR spectrophotometer was used to record the IR spectra of the synthesized MNF@mSiO_2_ at each step of the synthesis procedure. Shimadzu UV1800 spectrophotometer was used to record the absorption spectra of synthesized MNF@mSiO_2_ with variable shell thickness of mesoporous silica. Dynamic light scattering studies for the determination of hydrodynamic diameter and colloidal nature of the MNF@mSiO_2_ was conducted with Nano ZS, Malvern instrument. Crystallographic phase determination and crystal size of the synthesized MNF@mSiO_2_ was determined using Regaku D/max-2500 diffractometer over the 2θ range from 10° to 80° at a rate of 4°/min using Cu-K radiation (λ = 1.54060 A°). FEI quanta FEG-200 high resolution scanning electron microscope was used to confirm the mesoporous silica coated nanoparticles formation. Varian 240 FS Inductively Coupled Plasma Optical Emission Spectrometry (ICP-OES) was applied for the determination of Fe^3+^ and Mn^2+^ contents in the synthesized MNF. Magnetic property of the prepared MNF was identified using Lakeshore VSM 7410 and Phantom MR imaging studies were performed in GE signaHDxT 1.5 T MRI scanner.
