# Author Correction: Effect of Mesoporous Nano Water Reservoir on MR Relaxivity

**DOI:** 10.1038/s41598-018-28859-z

**Published:** 2018-07-12

**Authors:** Palani Sharmiladevi, Viswanathan Haribabu, Koyeli Girigoswami, Abubacker Sulaiman Farook, Agnishwar Girigoswami

**Affiliations:** 1 Faculty of Allied Health Sciences, Chettinad Hospital and Research Institute (CHRI), Chettinad Academy of Research & Education (CARE), Kelambakkam, Chennai, 603 103 India; 2 Department of Radiology, Chettinad Hospital and Research Institute (CHRI), Kelambakkam, Chennai, 603 103 India

Correction to: *Scientific Reports* 10.1038/s41598-017-11710-2, published online 11 September 2017

Some methodological details were omitted. In Results section, under the subheading ‘Determination of MR relaxivities’,

“The T1 relaxation for MNF and MNF@mSiO2 was determined as 425 ms and 138 ms respectively. On the other hand, the T2 relaxation was determined to be 75 ms and 35 ms for MNF and MNF@mSiO2 respectively.”

should read:

“The T1 relaxation for MNF and MNF@mSiO2 was determined as 425 ms and 138 ms respectively using images acquired from T1 FLAIR sequences with variable inversion recovery points. On the other hand, the T2 relaxation was determined to be 75 ms and 35 ms for MNF and MNF@mSiO2 respectively using T2 TSE sequence with variable TE.”

